# Effect of statins on coronary blood flow after percutaneous coronary intervention in patients with stable coronary artery disease

**DOI:** 10.1007/s12471-016-0883-x

**Published:** 2016-08-25

**Authors:** L. Cerit, H. Duygu, K. Gulsen, A. Gunsel

**Affiliations:** Department of Cardiology, Near East University Hospital, Nicosia, Cyprus

**Keywords:** Statin, Coronary blood flow, Stable coronary artery disease

## Abstract

**Aims:**

Statins have favourable effects on the vascular system. However, few data are available regarding the effect of these drugs on patients undergoing percutaneous coronary intervention (PCI). We sought to determine the impact of prior statin use on coronary blood flow after PCI in patients with stable coronary artery disease (CAD) by using the corrected thrombolysis in myocardial infarction (TIMI) frame count (CTFC).

**Methods:**

A total of 80 consecutive eligible patients (mean age: 60 ± 7 years, 65 % male) with the diagnosis of stable CAD who were hospitalised for elective PCI were retrospectively enrolled in our study. The study population was divided into two groups according to statin use at least 6 months before PCI. Group 1 comprised of 51 patients (67 % male; mean age: 58 ± 4 years) taking statins and group 2 comprised of 29 patients (62 % male; mean age: 60 ± 3 years) not taking statins. PCI was applied to de novo type A lesions. CTFC was calculated for the treated vessels at baseline and after PCI.

**Results:**

The two groups had similar characteristics in terms of age, sex, concomitant medications, lesion characteristics, pre-procedural CTFC, lipid parameters, and risk factors for CAD. Post-PCI CTFC (16 ± 3 vs. 22 ± 5, *p* = 0.01) and hs-CRP (2.1 ± 0.7 mg/l vs. 6.1 ± 2 mg/l, *p* = 0.01) in patients receiving statins before PCI were significantly lower than in patients without statin therapy. Multiple logistic regression analysis showed that statin pre-treatment (OR 2.5, 95 % CI 1.2 to 3.8, *p* < 0.001) and hs-CRP level (OR 1.8, 95 % CI 1.2 to 2.4, *p* = 0.001) were independent predictors of post-PCI CTFC.

**Conclusions:**

In patients with stable CAD undergoing PCI, receipt of long-term statin therapy was associated with improvement in epicardial perfusion after PCI.

## Introduction

Cholesterol reduction with HMG-CoA (3-hydroxy-3-methylglutaryl coenzyme A) reductase inhibitors or statins has been shown to improve mortality and cardiovascular morbidity in patients with established coronary artery disease (CAD) [1, 2]. Recent studies suggest that the incidence of early death and recurrent ischaemic events is also reduced by statin therapy in acute coronary syndrome (ACS) patients [3, 4]. However, few data are available regarding the effect of these drugs on patients undergoing percutaneous coronary intervention (PCI). Previous evidence suggests that statins have various favourable effects on the vascular system that are not directly related to their impact on lipid metabolism. Beyond lowering lipids, statins have favourable effects on platelet adhesion, thrombosis, endothelial function, plaque stability, and inflammation [5–8]. As with ACS, the vascular injury from coronary angioplasty and stent placement induces platelet activation, thrombosis, and inflammation within the vessel wall and the distal microvasculature. Therefore, in addition to a long-term benefit associated with lipid lowering, statin therapy might play a beneficial role early after PCI.

The thrombolysis in myocardial infarction (TIMI) frame count (TFC) is a simple clinical tool for assessing quantitative indexes of coronary blood flow. The TFC is objective, quantitative, reproducible, and sensitive to changes in coronary flow [9]. It has been suggested that a higher TFC may reflect disordered resistance vessel function or microvascular dysfunction [9]. To our knowledge, there are no reports regarding the effect of prior statin use on coronary blood flow in patients with stable CAD undergoing PCI. We sought to determine the impact of prior statin use on coronary blood flow after PCI in patients with stable CAD by using TFC.

## Methods

### Study population

A total of 80 consecutive eligible patients (mean age: 60 ± 7 years, 65 % male) with the diagnosis of stable CAD and who were admitted for elective PCI were retrospectively included in our study. The study population was divided into two groups according to statin use at least for 6 months before PCI. Group 1 comprised 51 patients (67 % male; mean age 58 ± 4 years) taking statins for at least 6 months before PCI and group 2 comprised 29 patients (62 % male; mean age: 60 ± 3 years) not taking statins. Statin treatment was started 10 ± 2 months prior to the PCI. The type and dosage of the statins used were as follows: (1) atorvastatin 79 % of cases (mean dose 30 ± 10 mg/day); (2) pravastatin, 11 % of cases (mean dose 20 ± 10 mg/day); and (3) simvastatin, 10 % of cases (mean dose 25 ± 10 mg/day).

PCI was performed based on the ongoing symptoms (refractory angina) despite optimal medical therapy. Chronic stable angina was defined as effort angina with objective ischaemic evidence on thallium scintigraphy or exercise testing over the past 6 months. Hypertension was defined as a blood pressure of 140/90 mm Hg or more or treatment with antihypertensive medication, current cigarette smoking as active smoking, and family history of CAD if patients had a first-degree male relative with evidence of CAD <55 years of age or a female relative <65 years of age. The body mass index was also measured. Exclusion criteria were: acute coronary syndromes; severe hepatic or renal dysfunction; diabetes mellitus; malignant or inflammatory diseases; local or systemic infection; anti-inflammatory drug use; and complex coronary lesions (total occlusions, highly calcified lesion, left main coronary artery lesion, restenosis, and vein graft lesion).

## Adjunctive pharmacotherapy

All patients received 100 mg per day of acetylsalicylic acid before intervention and unfractionated heparin during PCI on a routine basis. Clopidogrel was given in accordance with the guidelines. The glycoprotein IIb/IIIa inhibitors were administered at the discretion of the operator. Unfractionated heparin bolus at 100 IU/kg (50–70 IU/kg, if glycoprotein IIb/IIIa receptor inhibitor is administered) was given to all patients and the periprocedural activated clotting time (ACT) was measured. Unfractionated heparin dosage was adjusted under ACT guidance (ACT in the range of 250–300 or 200–250 s, if glycoprotein IIb/IIIa receptor inhibitors are given).

## Angiographic analysis

Selective coronary angiographic examinations were performed by the standard Judkins technique. The coronary diameters and stenosis percentages were measured by computerised quantitative angiography in a biplane mode. Angiographic assessment was always performed by two independent angiographers blinded to the patients’ clinical data.

Only bare-metal stents with or without balloon predilatation were used in this study. PCI was considered successful if the final percent diameter stenosis was <50 %, with TIMI grade 3 flow in the absence of recurrent ischaemia, myocardial infarction, need for bailout stenting or urgent coronary bypass surgery during hospitalisation, or death. Digital angiograms were then analysed by two independent, experienced interventional cardiologists, blinded to the data. All angiograms were assessed with respect to TIMI flow grade and TFC for the vessel in which the intervention was performed at baseline and after PCI.

## Determination of TIMI flow grade and TIMI frame count

TIMI flow grades have been described previously as grade 0, 1, 2, and 3 [10]. Coronary flow rates of all subjects were documented by TFC for each major coronary artery included in the study according to the method first described by Gibson et al. [9]. The left anterior descending coronary artery (LAD) is usually longer than the other major coronary arteries and the TFC for this vessel is often higher. To obtain a corrected TFC (CTFC) for the LAD, the TFC was divided by 1.7. Intraobserver and interobserver variability for CTFC was 1.3 ± 0.4 and 2.1 ± 0.6 frames, respectively.

## Biochemical analyses

Fasting blood samples were taken from all patients in the morning of the intervention day. Total cholesterol, triglyceride, high-density lipoprotein and low-density lipoprotein (LDL) levels were also measured by an auto analyser. Concentrations of high-sensitivity C‑reactive protein (hs-CRP) were measured by latex-enhanced immunoturbidimetry.

## Statistical analysis

Statistical analysis was performed using the SPSS statistical package (SPSS, Inc., Chicago, Illinois). Continuous variables were expressed as mean ± standard deviation and categorical variables as %. While the chi-square test or Fisher’s exact test was used for categorical values between the two groups, differences between groups in normally and non-normally distributed variables were assessed using the unpaired Student’s t test and the Mann-Whitney U test, respectively. Analysis of covariance (ANCOVA) was used to analyse the confounding effects of variables on the comparisons of the groups according to statin use before PCI. The multivariate analysis was used to define independent factors influencing CTFC after PCI. The following selected variables were inserted into the multivariate analysis: statin pre-treatment, age, body mass index, family history of CAD, systolic and diastolic blood pressure, hs-CRP level, hypertension, concomitant medications, smoking, serum total cholesterol, LDL-cholesterol levels, lesion length, residual percent stenosis, vessel diameter, and vessel type. A *p* value of <0.05 was considered statistically significant.

## Results

The characteristics of the study population are listed in Table 1. There was no statistically significant difference in the baseline characteristics analysed, which included patient age, sex, body mass index, left ventricular ejection fraction, systolic and diastolic blood pressure, hypertension, smoking, family history of CAD and various medication used. Lipid parameters were also comparable between the two groups (Table 1).

**Table 1:** Characteristics of the patients enrolled in the two groups

	Statin group (*n* = 51)	Control group (*n* = 29)	*p*-value
Age (years)	58±4	60±3	0.3
Male gender, *n* (%)	34 (67)	18 (62)	0.1
Left ventricular ejection fraction, %	65±8	65±5	0.7
Current smokers, *n* (%)	17 (33)	12 (41)	0.5
Systemic hypertension, *n* (%)	26 (50)	16 (55)	0.1
Body mass index, kg/m²	23±4	25±4	0.2
Family history of CAD	24 (47)	14 (48)	0.4
Systolic BP, mm Hg	120±5	125±10	0.1
Diastolic BP, mm Hg Heart rate, bpm	70±5 76±6	75±10 78±8	0.9 02
Number of diseased vessels, *n* (%) 1 2 3	– 34 (67) 12 (23) 5 (10)	– 17 (58) 9 (31) 3 (11)	– 0.9 0.1 0.4
Target vessel, *n* (%) LAD RCA LCX	– 25 (49) 18 (35) 8 (16)	– 14 (48) 10 (34) 5 (18)	– 0.2 0.1 0.8
Reference vessel diameter (mm)	3.0±0.1	3.2±0.2	0.2
Target vessel stenosis, %	80±10	85±10	0.09
Stent utilisation, *n* (%)	51 (100)	29 (100)	0.4
Stent diameter (mm)	3.4±0.2	3.5±0.3	0.1
Stent length (mm)	16±4	18±5	0.6
Dissection after PCI	0 (0)	0 (0)	1.0
Residual stenosis, %	15±5	20±5	0.3
Activated clotting time (s)	280±10	275±5	0.5
Pre-PCI CTFC (frames/s)	32±6	35±5	0.6
Post-PCI CTFC (frames/s) Post-PCI heart rate, bpm Post-PCI systolic BP, mm Hg Post-PCI diastolic BP, mm Hg	16±3 82±6 125±10 75±5	22±5 80±8 120±10 80±5	0.01 0.2 0.1 0.6
Total cholesterol (mmol/l)	4.3±0.2	4.1±0.3	0.1
LDL cholesterol (mmol/l)	2.4±0.6	2.2±0.5	0.1
HDL cholesterol (mmol/l)	1.1±0.3	1.0±0.2	0.4
Triglycerides (mmol/l)	1.5±0.2	1.5±0.2	0.6
hs-CRP (mg/l)	2.1±0.7	6.1±2	0.01
Medical therapy, *n* (%) Nitrates ACE inhibitors Beta-blockers Calcium antagonists Aspirin	– 51(100) 14 (27) 46 (90) 5 (10) 51 (100)	– 29(100) 8 (28) 25 (86) 4 (14) 29 (100)	– 1.0 0.7 0.1 0.4 1.0
Glycoprotein IIb/IIIa receptor blocker administration (tirofiban), *n* (%)	4 (7)	3 (10)	0.8

*CAD* coronary artery disease; *BP* blood pressure; *LAD* left anterior descending artery; *RCA* right coronary artery; *LCX* left circumflex artery; *PCI* percutaneous coronary intervention; *CTFC* corrected TIMI frame count; *LDL* low-density lipoprotein; HDL high-density lipoprotein; *ACE* angiotensin-converting enzyme*; hs-CRP* high-sensitivity C‑reactive protein

All the angiographic and procedural characteristics were similar in the two groups (Table 1). A final TIMI 3 flow was achieved in all patients. Angiographic complications during the procedure did not occur. It reached the target ACT levels during PCI in all patients. Glycoprotein IIb/IIIa inhibitors were administered in 4 of 51 patients (7.0 %) in the statin group and in 3 of 29 patients (10 %) in the control group (*p* = 0.8). Intracoronary vasodilator agents were not used.

**Fig. 1:**
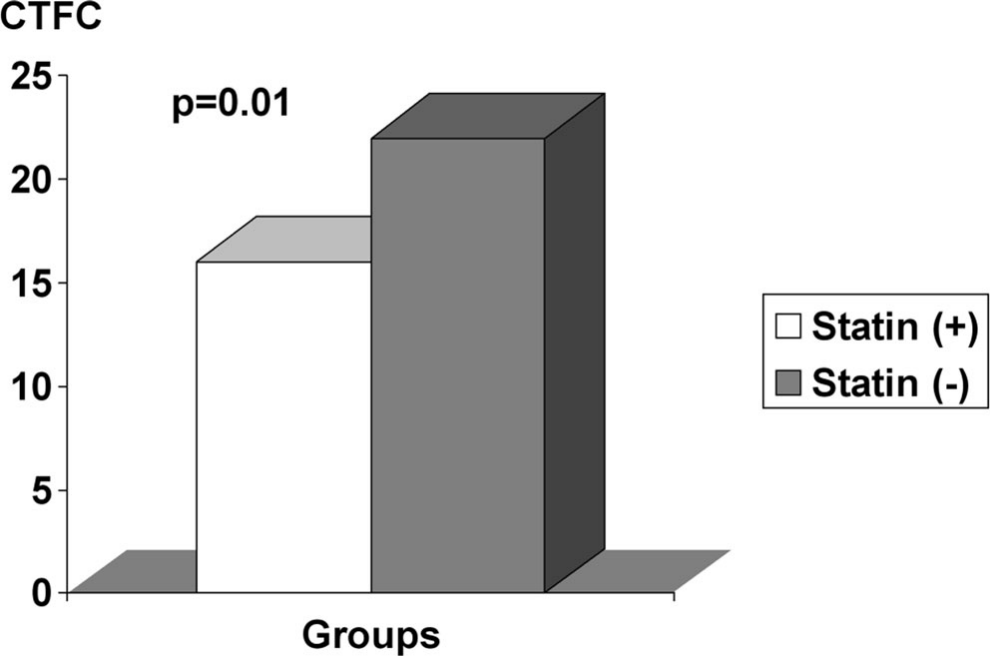
The comparison of corrected TIMI frame count (CTFC) between two groups

Although pre-PCI CTFC values were similar between the two groups (32 ± 6 vs. 35 ± 5, *p* = 0.6), post-PCI CTFC in patients treated with statin before PCI was significantly lower than the control group (16 ± 3 vs. 22 ± 5, *p* = 0.01, Fig. 1). The hs-CRP level was significantly lower in the patients taking statin in compared with the control group (2.1 ± 0.7 mg/l vs. 6.1 ± 2 mg/l, *p* = 0.01) (Table 1). No significant differences were found between the two groups for any of the other analysed variables. Multiple logistic regression analysis showed that only statin pre-treatment (OR 2.5, 95 % CI 1.2 to 3.8, *p* < 0.001) and hs-CRP level (OR 1.8, 95 % CI 1.2 to 2.4, *p* = 0.001) were independent predictors of post-PCI CTFC.

## Discussion

This study showed that receipt of chronic statin therapy before PCI in patients with stable CAD is associated with decreased CTFC of the target vessel, suggesting the improvement of microvascular function.

Conventional TIMI flow grading is a predictor of cardiac outcome after acute myocardial infarction and PCI, but it has several limitations [11]. The CTFC, another approach to grade flow impairment, is an objective, quantitative, reproducible, and sensitive index for coronary blood flow [9]. As indicated in our study, TIMI flow may appear normal visually, but may correlate to abnormal CTFC. The CTFC has been proposed to have incremental prognostic accuracy in predicting survival outcome with reperfusion therapy [12]. This measurement was significantly correlated with flow velocity measured with FloWire by several investigators during baseline conditions or hyperaemia [13]. So the CTFC may be an index of microvascular behaviour, which reflects coronary vascular resistance [14]. Higher CTFC values after PCI have also been found to be associated with poor clinical outcomes [15]. In addition to these studies using thrombolysis or balloon angioplasty, in which higher values of CTFC were associated with adverse clinical outcome, there are several studies regarding CTFC’s relevance in the current stent era [16–19]. In this study, the post-PCI CTFC in patients receiving statin before PCI was significantly lower than those of the patients not receiving statins. This finding may be attributed to the potential beneficial effects of statins on coronary microcirculation.

Lipid profiles were similar between the two groups in our study; therefore, the beneficial effects observed may be considered independent of cholesterol lowering. Although the present study was not designed to elucidate the potential mechanisms of the cardioprotective effect of the statins, some possible mechanisms can be hypothesised. It is suggested that improvement of myocardial vascular function by statins is not only a result of reduction in plasma lipids but is also caused by some action on blood vessels other than a lipid-lowering effect (so-called pleiotropic effects) [5]. The pleiotropic effects encompass non-lipid mechanisms that modify endothelial function, inflammation responses, plaque stability, and thrombus formation [20, 21]. Among those effects, improvement in endothelial function, anti-thrombotic and anti-inflammatory actions may be crucial for restoration of coronary blood flow after PCI, because those effects are consistent with a possible underlying mechanism of slow coronary flow [22]. As in coronary instability, vascular injury during PCI is associated with a systemically measurable inflammatory response and the degree of inflammation has been shown to be correlated with cardiovascular risks [23, 24]. The improvement of coronary blood flow associated with statin therapy might be explained based on its anti-inflammatory effects, but with the present evidence, this remains speculative.

Egashira et al. [25] demonstrated the improvement of endothelium-dependent coronary vasomotion using acetylcholine-stressed quantitative coronary arteriography and coronary flow velocity reserve using an intracoronary Doppler catheter after 2 and 6 months of pravastatin treatment in patients with dyslipidaemia. They speculated that impaired endothelium-dependent vasomotion may play a role in modulating myocardial perfusion in patients with hypercholesterolaemia, typical-effort angina pectoris, and critical stenosis of the epicardial coronary artery. It was also found that pravastatin increased microvascular perfusion in normocholesterolaemic patients with single-vessel disease after successful PCI [26]. Briguori et al. [27] showed that atorvastatin 80 mg loaded 24 hours prior to PCI in statin-naive patients significantly reduced the risk of periprocedural myocardial infarction as defined by elevations of creatine-kinase myocardial enzyme and cardiac troponin I. At 30 days, high-dose statin therapy reduced the risk of periprocedural myocardial infarction by 44 %, defined by both markers. The findings from NAPLES II support the need to postpone the intervention in statin-naive patients undergoing an elective procedure. These findings may be attributed to the potential beneficial effects of statins on CBF after PCI.

## Limitations

There are several limitations of this study. First, it was a retrospective study with a small number of patients. Larger multicentre studies are needed to corroborate our findings. Secondly, we could not estimate differences in efficacy among the various statins because of the relatively small numbers of patients in the study. There were substantial differences in CTFC pre- and post-PCI in this study. This would indicate that resting flow was impaired prior to PCI. Resting flow usually remains constant up to subtotal lesions. A proximal stenosis must generally exceed 75 % diameter narrowing before its resistance approximates that of the resting coronary vascular bed resistance and begins to reduce resting flow. In our study population, mean percent diameter stenosis was 80 and 85 % respectively. This might indicate that resting flow was impaired prior to PCI. The final limitation was the impossibility of follow-up, which could have informed us concerning the long-term clinical outcomes.

## Conclusions

In conclusion, the present study showed that statin therapy before PCI in patients with stable CAD may have some beneficial effects on coronary blood flow demonstrated by CTFC. Further large-scale prospective randomised clinical trials are needed to elucidate the underlying mechanisms and clinical importance of these findings.
